# EFFECTS OF MEDICAID MANAGED CARE ENROLLMENT ON HOSPITALIZATION RISK AMONG DUAL-ELIGIBLES

**DOI:** 10.1093/geroni/igz038.2019

**Published:** 2019-11-08

**Authors:** Rebecca Gorges

**Affiliations:** University of Chicago, Chicago, Illinois, United States

## Abstract

Many state Medicaid agencies have recently expanded Medicaid managed care (MMC) to include low-income older adults that are Medicare-Medicaid dually enrolled (duals). While little evidence exists regarding the effects of these expansions on health outcomes, duals may be especially vulnerable to low-quality or low-intensity care delivered by MMC due to their high use of services and the fragmentation inherent in the two-payer system. Using difference-in-differences (DID) and instrumental variables estimation, this study provides the first national examination of the impact of MMC on hospitalization for duals using claims data. DID results indicate that managed long-term services and supports (LTSS) expansions among duals are associated with increased rates of hospitalization while comprehensive managed care results in no change in hospital use. Further analyses exploring heterogeneity shed light on how these managed care payment models affect older adults and individuals using LTSS.

